# Dinner to Dr. Randolph

**Published:** 1842-09-10

**Authors:** 


### MEDICAL EXAMINER.

NEW SERIES.

No. 37.] PHILADELPHIA, SEPTEMBER 10, 1842. [Vol. I.

### DINNER TO DR. RANDOLPH.

On Wednesday last a very large number of the professional friends of Dr. Jacob Randolph took occasion to welcome him with a public dinner, upon his recent return from Europe—a well merited compliment, due alike to the distinguished professional position, character, and personal qualities of Dr. Randolph.

From the list of names appended to the letter which we publish below, it will be seen that the great body of the profession of Philadelphia, worthily headed by the venerable Chapman, united in this tribute of respect and regard.

[*Correspondence.*]

Philadelphia, 24th Aug., 1842.

Dear Sir,—Entertaining the greatest respect for your character and professional attainments and services, your medical friends are desirous to give some manifestation of this feeling towards you, and, at the same time, cordially to welcome your return to them and your country. They, therefore, beg of you to accept of a dinner, on any day, after the first of September next, that may be most convenient to you. Trusting that you will not disappoint their wishes, the undersigned have great pleasure in subscribing themselves,

Very faithfully, your friends,

N. Chapman, Thos. Harris, F. Bache, Wm. Rush, Henry Neill, John Bell, Saml. Geo. Morton, W. W. Gerhard, Francis West, jr., Edward Peace, W. H. Gillingham, J. B. Biddle, M. Clymer, G. Emerson, Samuel McClellan, Robley Dunglison, Henry H. Smith, Joseph Peace, William Harris, Joseph G. Nancrede, W. S. W. Ruschenberger, B. H. Coates, H. L. Hodge, J. K. Mitchell, R. M. Huston, W. D. Brincklé, T. R. Colhoun, Isaac Hays, Joseph Pancoast, W. Poyntell Johnston, T. R. Brincklé, S. Jackson, D. H. Tucker, Paul B. Goddard, Wm. Byrd Page, D. C. Skerrett, Jno. Rodman Paul, J. F. Meigs, George W. Norris, William Pepper, J. Rhea Barton, W. E. Horner, R. M. Patterson, D. P. Lajus, Thomas F. Betton, M. M. Reeve, Thomas Dillard, G. Moehring, Thomas D. Mütter, J. M. Wallace, George Fox.

*To J. Randolph, M. D.*

—

Philadelphia, Aug. 24, 1842.

Gentlemen,—I had the honour of receiving, this morning, your very kind and complimentary letter, containing an invitation to a dinner, to be given me by some of my medical friends. Although I cannot possibly conceive that I am at all entitled to such a distinguished mark of your approbation, still my feelings will not permit me to decline an invitation so flattering to myself, and coming from those for whom I have so long cherished the most sincere private and professional friendship and regard.

It will give me great pleasure to dine with you upon any day after the 1st of September, which will best suit your convenience.

With sentiments of the highest esteem,

I am very sincerely,

Your friend and servant,

J. RANDOLPH.

To Drs. N. Chapman, Thomas Harris, Franklin Bache, *&c. &c. &c.*

We had the pleasure of participating in the very agreeable festivities of the occasion. Dr. CHAPMAN presided, assisted by Dr. JACKSON, Dr. FRANKLIN BACHE, and Dr. WILLIAM RUSH, as Vice Presidents. It is hardly necessary to say, that the utmost cordiality and good fellowship prevailed at the entertainment. The eminent social qualities of the distinguished presiding officer were never displayed with happier effect, and the flow of soul—of wit and good feeling—in which he brilliantly led the way, appeared to be *epidemically* diffused throughout the company. In a speech of great eloquence and feeling, which was warmly and heartily responded to by his hearers, Dr. CHAPMAN expressed the common sentiments of the assemblage towards their guest, to whom, in apposite language, he paid the combined tribute of respect and esteem which he has so well earned professionally and individually. Dr. RANDOLPH returned thanks neatly and effectively.

Our limits deny us the pleasure of enumerating the many good speeches which were made on the occasion, but we must be allowed briefly to allude to one of the pleasantest incidents of the evening—a poetical effusion, concontributed by our friend, Dr. B. H. COATES. This was sung with much applause—a happy proof that high professional attainments and extensive occupation are not incompatible with the successful cultivation of the lighter field of fancy.

